# Oocyte aging in comparison to stem cells in mice

**DOI:** 10.3389/fragi.2023.1158510

**Published:** 2023-04-11

**Authors:** Go Nagamatsu

**Affiliations:** ^1^ Center for Advanced Assisted Reproductive Technologies, University of Yamanashi, Kofu, Yamanashi, Japan; ^2^ Precursory Research for Embryonic Science and Technology, Japan Science and Technology Agency, Kawaguchi, Saitama, Japan

**Keywords:** germ cells, oocyte, dormancy, stem cells, aging, *in vitro* culture

## Abstract

To maintain homeostasis, many tissues contain stem cells that can self-renew and differentiate. Based on these functions, stem cells can reconstitute the tissue even after injury. In reproductive organs, testes have spermatogonial stem cells that generate sperm in men throughout their lifetime. However, in the ovary, oocytes enter meiosis at the embryonic stage and maintain sustainable oogenesis in the absence of stem cells. After birth, oocytes are maintained in a dormant state in the primordial follicle, which is the most premature follicle in the ovary, and some are activated to form mature oocytes. Thus, regulation of dormancy and activation of primordial follicles is critical for a sustainable ovulatory cycle and is directly related to the female reproductive cycle. However, oocyte storage is insufficient to maintain a lifelong ovulation cycle. Therefore, the ovary is one of the earliest organs to be involved in aging. Although stem cells are capable of proliferation, they typically exhibit slow cycling or dormancy. Therefore, there are some supposed similarities with oocytes in primordial follicles, not only in their steady state but also during aging. This review aims to summarise the sustainability of oogenesis and aging phenotypes compared to tissue stem cells. Finally, it focuses on the recent breakthroughs *in vitro* culture and discusses future prospects.

## 1 Introduction

Germ cells are the only cell type that transmit genetic information to the next-generation (Kurimoto and Saitou, 2019). Oocytes are important cells responsible for embryogenesis. Advances in somatic cell cloning technology have allowed the creation of an organism even from somatic cells (Wakayama et al., 2008), as long as the genome remains; however, an oocyte is necessary to carry out development. In mammals, since the oogonia enter meiosis in the embryonic ovary by retinoic acid signal (Bowles et al., 2006), oocyte numbers are limited after birth. As a result, non-proliferating oocytes are maintained in primordial follicles. Furthermore, the sustainable ovulatory cycle is maintained by activating some oocytes, while others maintain a dormant state (Nagamatsu, 2021). Oocytes originate from primordial germ cells (PGCs), which are originated from the epiblast of the early embryo. PGCs proliferate in the foetal ovary and form cyst structures connected by intercellular bridges formed by incomplete cytokinesis (Soygur et al., 2021). In the cyst, the oocyte enters meiosis and is arrested at prophase I. Therefore, the number of oocytes does not increase thereafter. Around birth, the cyst structure is broken down, and the oocyte forms a follicle surrounded by granulosa cells (Grive and Freiman, 2015). The most immature follicle is the primordial follicle, which is composed of an oocyte less than 20 μm in diameter and a single layer of flattened granulosa cells (Pedersen and Peters, 1968). Nuclear localization of forkhead box O3 (FOXO3) is critical for maintaining oocyte dormancy in the primordial follicle (Castrillon et al., 2003). When c-Kit on oocytes is activated by stem cell factor (SCF) from granulosa cells, a downstream signal phosphorylates FOXO3, which translocates to the cytoplasm and induces oocyte maturation (Jones and Pepling, 2013). The balance between dormancy and activation is directly related to duration of reproductive life, and its disruption causes primary ovarian insufficiency (POI) (Barros et al., 2020).

While oocytes adopt such a unique homeostatic mechanism, many other tissues use stem cell systems. Stem cells can self-renew and differentiate, maintain homeostasis, and reconstitute tissues when damaged (Fuchs and Chen, 2012). For example, skin stem cells support skin metabolism by producing functionally differentiated cells, such as keratinocytes, melanocytes, and fibroblasts, while maintaining themselves through self-renewal (Wang et al., 2016). As self-renewal of stem cells is rare, there are some similarities in the regulatory mechanisms of oocytes in primordial follicles. For example, in order to avoid DNA damage caused by the generation of reactive oxygen species (ROS), cells are placed in a hypoxic environment (Simon and Keith, 2008).

The ovary is one of the earliest organs in the body to undergo aging because the number of oocytes stored in the ovary is not sufficient for lifelong oogenesis. After menopause, the ovaries cease hormone secretion, and various effects occur, collectively known as climacteric disorder (Mangione et al., 2022). Furthermore, before menopause, decline in oocyte function accompanies the decrease in their numbers with aging. In aged oocytes, chromosome aneuploidy increases and mitochondrial function declines (Wu et al., 2022). Meanwhile, the number of tissue stem cells also decreases with aging, and it could be hypothesized that oocytes and stem cells share common aging phenotypes.

Female germline stem cells have been extensively studied in *Drosophila*. In 2004 (Hinnant et al., 2020), the existence of oocyte stem cells in mice was reported (Johnson et al., 2004). Since then, there have been researches on both supporting and pointing out the misinterpretation and extensive debates are ongoing (Horan and Williams, 2017; Wagner et al., 2020; Alberico et al., 2022). This review focuses on the quiescent oocyte in the primordial follicle. Then, the aging of oocytes is summarized in comparison to that of stem cells, and future directions for elucidating the mechanism of oocyte aging are discussed, including the application of recently developed *in vitro* culture techniques.

## 2 Oocyte development

Mammalian oocytes originate from PGCs, whose differentiation is specified soon after implantation (Cantú and Laird, 2013). PGCs migrate to embryonic gonads and differentiate further according to sex. In the female gonads, PGCs proliferate with incomplete cytokinesis and form germline cysts connected by intracellular bridges (Greenbaum et al., 2011). Then, they enter meiosis synchronously and become arrested at prophase I (Spiller and Bowles, 2022). Around birth, germ cell cysts are broken down to generate follicles composed of oocytes surrounded by granulosa cells (Kerr et al., 2013). At the time of cyst breakdown, many oocytes undergo apoptosis due to an unclarified mechanism called foetal oocyte attrition (FOA). At the time of FOA, the repression of transposable elements is reportedly important for oocyte survival (Malki et al., 2014). Taken together, since oocytes not only stop proliferation at birth but also decrease in number, they must maintain a sustainable ovulatory cycle by a limited number (Kerr et al., 2013).

**FIGURE 1:**
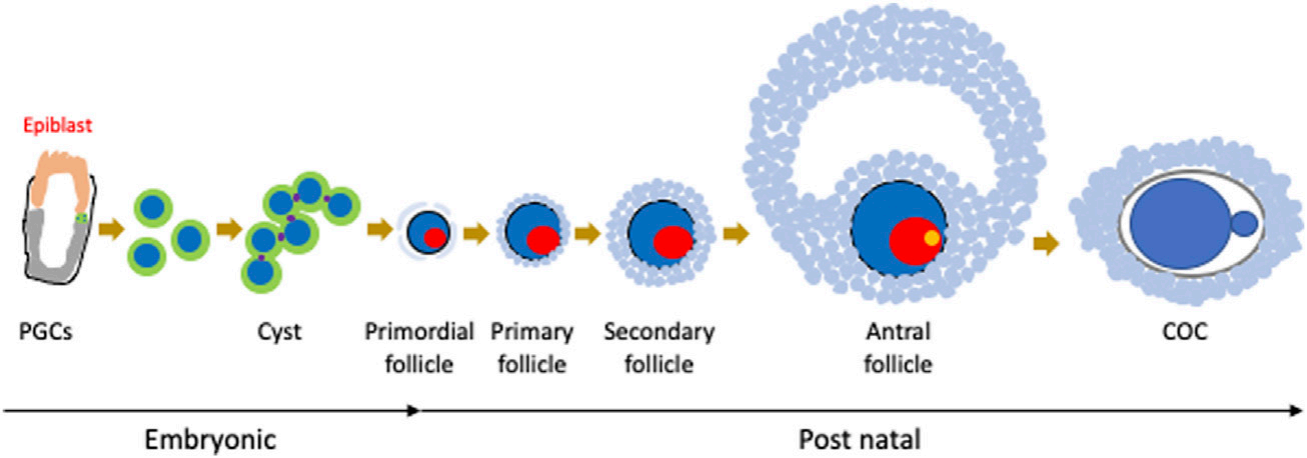
Oocyte development in mice. As the origin of oogenesis, primordial germ cells (PGCs) are specified from epiblasts, migrate to the embryonic ovary, form cysts, and enter meiosis. After birth, cysts are broken down and oocytes form follicles with granulosa cells. Follicles then mature as an enlarging oocyte mass and proliferation of granulosa cells. Finally, oocytes are ovulated as cumulus oocyte complexes (COCs).

Oogenesis starts from primordial follicles, characterised by small oocytes and a single layer of flat-shaped granulosa cells (Nagamatsu, 2021). Follicle maturation proceeds by enlarging oocyte size and proliferation of granulosa cells. Initially, granulosa cells change shape from flat to cuboidal and proliferate to form a multilayer around the oocyte and finally form the follicular cavity. At that time, the oocytes have large nuclei called germinal vesicles. Granulosa cell proliferation is regulated by bone morphogenetic protein (BMP) 15 and growth differentiation factor (GDF) 9, which are secreted by oocytes (Dong et al., 1996). In antral follicles, oocyte maturation is characterised by germinal vesicle breakdown following stimulation with follicle stimulating hormone (FSH) and luteinizing hormone (LH). Oocytes resume meiosis and complete division I, then stop again at metaphase II. The oocyte is then ovulated, and entry of sperm triggers the completion of the second division of meiosis (Figure 1). At metaphase I, chromosome segregation errors can result in aneuploidy in the embryo (Hassold et al., 2007). As most trisomies and autosomal monosomies are inviable, the regulation of chromosome segregation in oocytes is important. To ensure this, cohesins that cohere sister chromatids and centrosomes play a critical role. In addition, during oocyte maturation, many proteins and organelles are stocked, especially mitochondria, which play a central role in the generation of ATP by oxidative phosphorylation (OXPHOS). A drastic increase in mitochondrial copy number occurs at the late stage of folliculogenesis (St. John, 2014). Approximately 100,000 mitochondria are stocked in fully grown oocytes (Cao et al., 2007; Wai et al., 2008); normal somatic cells have approximately 100–10,000 mitochondria, oocytes have 10–1,000 times more. The amount of mitochondrial DNA remains the same between metaphase II (MII) oocytes and hatched blastocysts (Thundathil et al., 2005; Chappel, 2013). Furthermore, the structure of the mitochondria also changes during oogenesis. While mitochondria are round-shaped with lamellar cristae in PGCs, they are elongated with numerous transversally oriented cristae in oocytes (Wassarman and Josefowicz, 1978; Motta et al., 2000; Trebichalská et al., 2021). The mitochondria continue to undergo fission and fusion to maintain homeostasis. Dynamin-related protein (DRP) 1 and mitofusin (MFN) 1 are key regulators of mitochondrial fission and fusion, respectively. Oocyte-specific DRP1 knockout (KO) mice with defective fission show insufficient oocyte maturation and infertility (Udagawa et al., 2014).

### 2.1 Primordial follicle dormancy and activation as a system to maintain sustainability apart from stem cells

**FIGURE 2:**
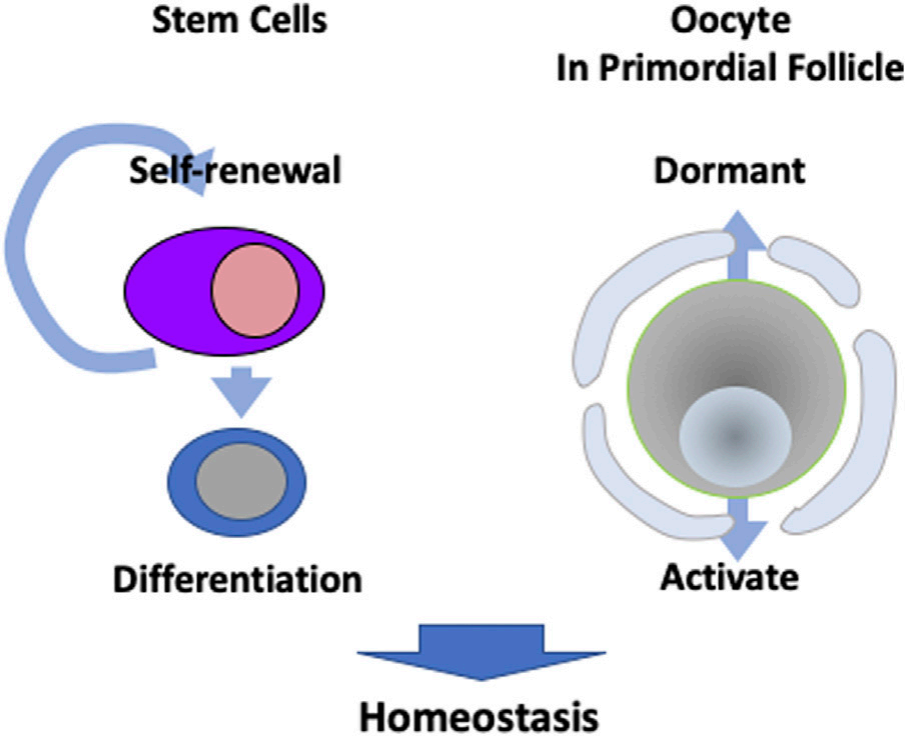
Different strategies to maintain homeostasis. Many tissues have stem cells that are capable of self-renewal and differentiation for metabolism. However, oocytes in primordial follicles are kept dormant, while a portion are activated in order to maintain sustainable ovulation cycles.

Stem cells play an important role in the sustainable activity of tissues through their ability to self-renew for functional cell maintenance and differentiation. Stem cells are characterised by these two abilities and can even reconstitute injured tissue. Among the reproductive organs, the testes have spermatogonial stem cells that generate sperm throughout a man’s life. However, in the ovary, oocytes enter meiosis at the embryonic stage and maintain oogenesis in the absence of stem cells. Oocytes in primordial follicles, which are the most immature oocytes in the ovary, are maintained in a dormant state and some are activated to form mature oocytes (Nagamatsu, 2021). The regulation of dormancy and activation is critical for a sustainable ovulatory cycle and is directly related to the female reproductive cycle (Figure 2).

The regulation of oocytes in primordial follicles has been well-studied in mice. A critical regulator of dormancy is FOXO3. FOXO3-deficient mice showed activation of all oocytes soon after birth, resulting in primary ovarian insufficiency (POI) (Castrillon et al., 2003). FOXO3 is regulated by its translocation from the nucleus to the cytoplasm, which restricts its transcriptional activity through phosphorylation by AKT. FOXO3 phosphorylation is triggered by SCF-mediated c-Kit activation (Jones and Pepling, 2013), especially tyrosine phosphorylation at position 719, which induces Phosphoinositide 3-kinase (PI3K) activation (Kissel, 2000). PI3K activates AKT through phosphoinositide-dependent kinase (PDK) 1. As phosphatase and tensin homolog (PTEN) has the opposite activity to PI3K, PTEN deficiency induces activation of dormant oocytes and results in POI (Reddy et al., 2008). Although forced expression of an active form of FOXO3 induced by mutation in the nuclear export signal in oocytes slows oocyte growth and follicle development, it does not maintain all oocytes in dormancy (Liu et al., 2007; Pelosi et al., 2013). Therefore, there are other mechanisms that maintain dormant oocytes.

### 2.2 Regulation of oocyte maintenance in primordial follicles compared to that in stem cells

Although stem cells have a proliferative capacity, their self-renewal is rare. Essentially, they remain in a resting state for a long time. Therefore, there are some similarities between oocytes in primordial follicles and stem cells. For example, FOXO3, an essential factor for the dormancy of oocytes in primordial follicles, is critical for stem cell regulation. In the haematopoietic system, FOXO3 deficiency causes cell cycle entry of quiescent haematopoietic stem cells (HSCs), resulting in the exhaustion of long-term repopulating capacity through the regulation of cell cycle suppressors, such as *p27* and *p57* (Miyamoto et al., 2007; Tothova et al., 2007). In addition, FOXO3 regulates HSC redox balance. Although FOXO3 KO HSCs exhibit upregulated ROS, they also show downregulation of superoxide dismutase (SOD) family genes and ataxia telangiectasia mutated (ATM), which act in the response to oxidative stress (Tothova et al., 2007). Furthermore, FOXO3 KO HSCs show defective mitochondrial aspiration (Rimmelé et al., 2015). FOXO3 also plays a critical role in neural stem cell (NSC) maintenance. FOXO3-deficient NSCs have decreased neurosphere-forming capacity (Yeo et al., 2013). It has been reported that FOXO3 regulates quiescence-related genes, such as *p27* and *CCNG2,* as well as ROS-detoxifying enzymes, such as peroxiredoxin (PRDX) and Sestrin 3 in NSCs (Paik et al., 2009; Renault et al., 2009). FOXO3-deficient muscle stem cells (MSCs) show an abnormal transition to a quiescent state after activation (Gopinath et al., 2014). Although the precise mechanism has not been analysed, studies show that FOXO3 targets miR-484, which regulates mitochondrial fission 1 (FIS1) in MSCs (Wang et al., 2012).

Although the mechanism by which FOXO3 maintains dormancy in oocytes is unclear, oocytes in primordial follicles reportedly prefer glycolysis to OXPHOS, similar to haematopoietic stem cells (Takubo et al., 2013; Shimamoto et al., 2019). In addition, because primordial follicles are located at a distance from the vasculature in the ovary (Feng et al., 2017), they may be in a hypoxic microenvironment, similar to haematopoietic stem cells. Furthermore, although oocytes in primordial follicles enter meiosis, research suggests that *p27* is a target of FOXO3 as well as stem cells. Thus, as oocytes in primordial follicles and stem cells are assumed to have similarities, mutual comparison is important for further understanding these mechanisms.

Some stem cells, such as intestinal stem cells (ISCs), actively proliferate to maintain homeostasis (Hageman et al., 2020). In ISCs, the classical Wnt signal is known to be the most important for their maintenance. Inactivation of Wnt leads to the disappearance of ISCs, while forced activation of Wnt leads to abnormal proliferation of stem cells, which indicates malignant transformation. Wnt signals are also used as proliferative signals in other stem cells, such as hematopoietic stem cells. It has been shown that non-canonical Wnt signals, not classical Wnt signals, are involved in the maintenance of resting phase (Sugimura et al., 2012). In primordial follicles, classical Wnt signals have been shown to act on granulosa cells and induce activation of primordial follicles (Habara et al., 2021). ISCs, which actively proliferate, use proliferation signals for their maintenance and are thought to have a different mechanism from resting stem cells and primordial follicles.

On the other hand, there are non-stem cells, such as pancreatic *ß* cells, that function without proliferating (Tudurí et al., 2022). When pancreatic *ß* cells secrete insulin, dopamine, which is a monoamine, is also released outside the cells simultaneously. The released dopamine then acts as feedback to suppress insulin secretion. Monoamines are known to generate reactive oxygen species (ROS) when metabolized by monoamine oxidase B (MAOB) inside the cells. Therefore, pancreatic *ß* cells have a mechanism to properly store dopamine inside cells, and if this mechanism fails, the production of ROS is increased, leading to cell dedifferentiation and cell death (Sakano et al., 2020).

Thus, despite differences in the proliferative capacity and stemness, the cells involved in homeostatic maintenance have to protect genome stability. Therefore, comparing the mechanisms such as ROS regulation are of interest for our understanding of homeostasis maintenance in aging.

## 3 Ovarian aging

Ovaries are one of the earliest organs to age because oocyte storage is insufficient to maintain a lifelong ovulation cycle. Owing to this shortage of oocytes, several defects occur, such as reproductive decline, endocrine dysfunction, and menstrual cycle abnormalities. Ovarian aging accelerates from age 35, and the average age of menopause is between 50 and 52 years (Mishra et al., 2019). Furthermore, for reasons such as genetics and chemotherapy, oocyte shortage at an early age leads to disrupted hormone secretion which is the same defects as aging, called POI. It has been reported that 1% of women under 40 years of age and 0.1% of women under 30 years of age suffer from POI for genetic reasons (Chon et al., 2021). Therefore, oocyte shortage is a major cause of ovarian ageing. Due to menopause, ovarian function declines, while oestrogen and progesterone secretion are suppressed, resulting in a hormonal imbalance that induces various symptoms collectively called climacteric disorders.

Recently, it was reported that ovarian fibrosis and stiffness increase with age (Amargant et al., 2020) because of an increase in hyaluronan materials and decreased collagen. Physical pressure is important for maintaining oocyte dormancy in primordial follicles (Nagamatsu et al., 2019). Experiments suggest that as the effects of pressure can be mimicked by stiffness (Smith et al., 2017), age-related ovarian fibrosis increases internal pressure. Intriguingly, there are cases where primordial follicles remain even during POI, and excision of the ovary *in vitro* can induce follicle activation (Vo and Kawamura, 2021). As intraovarian pressure control has the potential to extend reproductive lifespan, further investigation is required.

## 4 Oocyte aging

Before menopause, oocyte function decreases with increasing age. There are several defects reported, such as chromosome miss-segregation, mitochondria dysfunction, and activation of retrotransposable elements. Among these, increased aneuploidy has been well-studied. As mis-segregation of chromosomes causes aneuploidy, sister chromatids must remain together until anaphase by cohesion (Chiang et al., 2011; Berkowitz et al., 2020). The prevalence of meiotic cohesins decreases with age (Duncan et al., 2012; Tsutsumi et al., 2014). Furthermore, aged oocytes are more susceptible to removal by separase, a cysteine protease that cleaves the key cohesion subunit, Rec8 (Uhlmann et al., 2000; Waizenegger et al., 2000). Shugoshin-like 2 (SGO2) levels also decrease with age (Lister et al., 2010; Rattani et al., 2013). SGO2 is critical for protecting centromeric cohesion; SGO2-deficient oocytes showed premature separation of sister chromatids (Rattani et al., 2013). Although mitochondria-coupled ATP production is essential for the resumption of meiosis, mitochondrial function decreases with age (Al-Zubaidi et al., 2019; Abbassi et al., 2021) and ATP production decreases in aged oocytes (Selesniemi et al., 2011). Furthermore, mitochondria in oocytes not only decrease in number, but also exhibit morphological abnormalities according to age (Simsek-Duran et al., 2013). Meanwhile, the expression of retrotransposons L1 and Intracisternal A-particle (IAP) increases in oocytes with age, and this is correlated with the increase in Rad51 and γH2AX foci, which are markers of DNA damage and repair (Wasserzug-Pash et al., 2022).

It is not clear whether aged oocyte dysfunction is due to the maturation process or the quality of primordial follicles. However, because of their shortage, it is difficult to study aged primordial follicles in detail. Recently, it was reported that the reconstitution of oogenesis and induction of primordial follicles can be performed *in vitro* (Hikabe et al., 2016; Nagamatsu et al., 2019). This *in vitro* culture system is expected to solve the problem of number and enable the analysis of age-related changes in primordial follicles, as described in the following section.

### 4.1 Comparison of oocyte and stem cell aging

**FIGURE 3:**
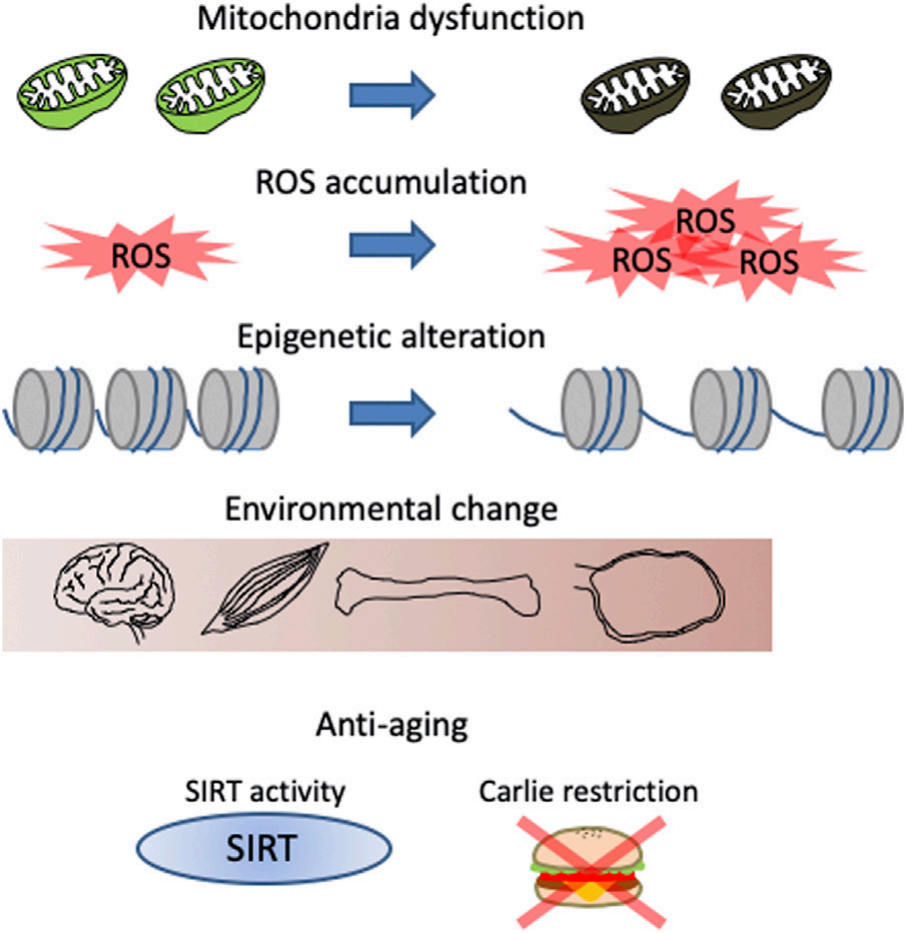
Features of aging phenotypes in both stem cells and oocytes in primordial follicles. Changes related to energy metabolism, such as mitochondrial dysfunction and reactive oxygen species (ROS) accumulation, changes in gene expression due to chromatin relaxation, and changes in the microenvironment such as fibrosis and changes secreted cytokines are known. Sirtuin (SIRT) activation and calorie restriction are known methods to delay these changes.

Similar to oocytes, stem cells also experience a decline in number and function during aging (Jejurikar et al., 2006; Yamakawa et al., 2020). During tissue homeostasis maintenance, stem cells accumulate DNA damage, altered epigenetic modifications, and reduced mitochondrial function. These factors are thought to cause the aging phenotype of stem cells and can likely be applied to oocytes (Figure 3). As described above, mitochondrial function in oocytes is known to decrease with age and mitochondrial damage accelerates aging in epidermal stem cells (Velarde et al., 2015). In HSCs, while the number of cells with low mitochondrial membrane potential (MMP) increases with age, Mito-q (a mitochondria-targeted antioxidant) treatment increases MMP and induces young phenotypes (Mansell et al., 2021). Thus, mitochondrial dysfunction is a common feature of both oocytes and stem cells in aging. Defective mitochondria in aged oocytes increase ROS levels (Wu et al., 2022). HSCs also exhibit increased ROS production during aging (Nakamura-Ishizu et al., 2020). Although ROS function as secondary messengers for cellular signalling, excessive ROS causes DNA damage, which is known as oxidative stress.

Aberrant DNA methylation in stem cells increases with age, a phenomenon known as epigenetic drift (Beerman et al., 2013; Hernando-Herraez et al., 2019). In addition to DNA methylation, histone modification is also affected by aging, especially the heterochromatin marks H3K9me2, H3K9me3, and H4K20me3 (Diao et al., 2021). In the Werner syndrome model of WRN deficiency, the global loss of H3K9me3 in MSCs drives aging phenotypes (Zhang et al., 2015). Although the precise mechanisms are still elusive, loss of heterochromatin is related to the reactivation of suppressed repeated DNA elements. In oocytes, heterochromatin histone marks H3K9me2 and H3K27me3 are lost in aged oocytes, and impaired silencing of retrotransposons and inhibition of these transcripts by azidothymidine partially ameliorates the maturation of aged oocytes (Wasserzug-Pash et al., 2022). In addition to methylation, acetylation influences stem cell aging. Recently, it was identified that lysine acetyltransferase 7 (KAT7), a histone acetyltransferase, is a senescence driver whose deletion leads to the deacetylation of H3K14 and repression of p15 (Wang et al., 2021). Although the function of KAT7 in oocytes has not been elucidated, studies show there is a gradual increase in H3K14ac levels during oocyte aging (Huang et al., 2007).

Microenvironmental changes also occur with age. In the ovary, fibrosis occurs with aging, as described above. Furthermore, recent research shows that gene expression in cumulus cells is altered earlier than that in oocytes (Mishina et al., 2021). In HSCs, aged bone marrow increases transforming growth factor (TGF) -β and interleukin (IL) -6 secretion, and inhibiting these signals restores HSCs function (Valletta et al., 2020). In hair follicle stem cells, an altered extracellular matrix correlates with a decline in their function with aging (Ge et al., 2020).

Calorie restriction prevents aging-associated functional decline in many cell types in diverse species (Benayoun et al., 2015; Zhang et al., 2020). In stem cells, for example, calorie restriction improves the repopulating capacity of HSCs (Tang et al., 2016), promotes intestinal stem cell (ISC) expansion (Igarashi and Guarente, 2016), and enhances skeletal MSC function by increasing the number of mitochondria (Cerletti et al., 2012). Moreover, during oocyte aging, calorie restriction prevents functional decline, including decreasing aneuploidy in mouse oocytes (Selesniemi et al., 2011). Consistent with this, calorie restriction also prevents spindle abnormalities and attenuates the reduction in cohesin levels (Selesniemi et al., 2011; Mishina et al., 2021).

Downstream of calorie restriction, sirtuin (SIRT), an nicotinamide adenine dinucleotide (NAD)^+^-dependent histone deacetylase, has been identified as a functional molecule that regulates aging (Imai et al., 2000). SIRT7 causes a delay in MSC aging, and its deficiency enhances senescence (Bi et al., 2020). In HSCs, Sirt3 and Sirt7 reportedly decrease with aging, and their overexpression ameliorates the aging phenotype (Fang et al., 2020; Kaiser et al., 2020). Since the phenotype of SIRT1 KO mice is controversial because of the genetic background of mice (McBurney et al., 2003; Li et al., 2007; Blagosklonny et al., 2008), its functions in oocyte aging have not been elucidated. However, since SIRT1-7 expression in oocytes was confirmed, further analyses are expected (Kawamura et al., 2010). Interestingly, research shows SIRT1 upregulation by resveratrol treatment prevents ovarian aging (Liu et al., 2013).

Not only similarities but also differences are existing. For example, lineage-biased differentiation is one of the aging phenotypes in hematopoietic stem cells (Haas et al., 2018). However, as oocytes in primordial follicles are the lineage restricted cells, the characteristic is not observed.

## 5 *In vitro* reconstitution of oogenesis

**FIGURE 4:**
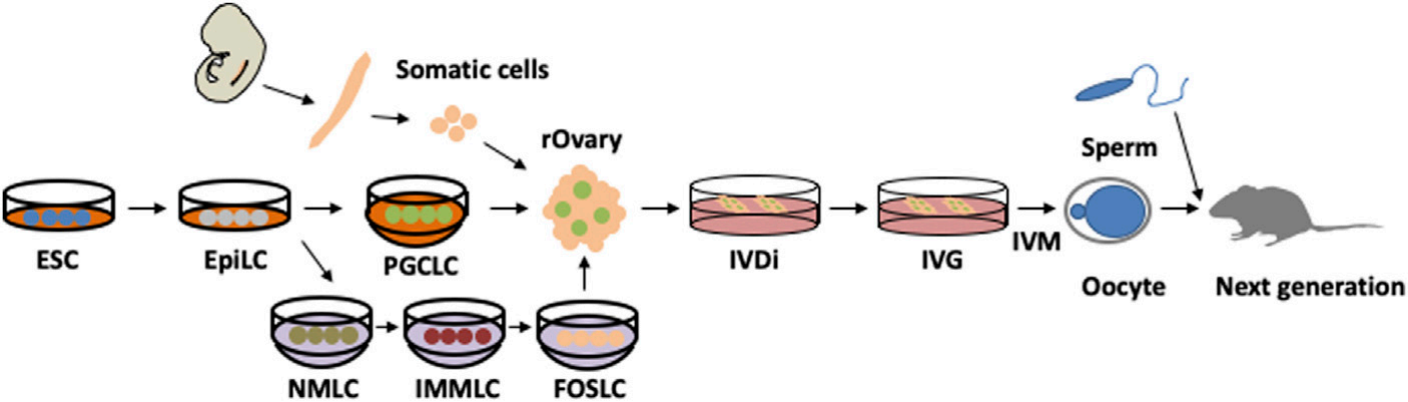
*In vitro* culture for entire oogenesis from pluripotent stem cells. Embryonic stem cells (ESCs) are induced to primordial germ cell-like cells (PGCLCs) *via* epiblast-like cells (EpiLCs) by mimicking *in vivo* development. PGCLCs are further differentiated in a recombinant ovary (rOvary) by aggregation with embryonic ovarian somatic cells. *In vitro* differentiation (IVD) generates secondary follicles, which further mature through *in vitro* growth (IVG). Then, cumulus oocyte complexes are picked up and cultured using *in vitro* maturation (IVM). After *in vitro* fertilization (IVF) and transfer to a surrogate mother, healthy offspring are generated. Recently, ovarian somatic cells were also induced from ESCs through EpiLC. First, nascent mesoderm-like cells (NMLCs) were induced from EpiLCs. NMLCs were differentiated to intermediate mesoderm-like cells (IMMLC) and finally induced into fetal ovarian somatic cell-like cells (FOSLCs). These processes are well traced within *in vivo* development.

Recently, *in vitro* culture for oogenesis has progressed drastically; it is now possible to reproduce oogenesis *in vitro* from PGCs in embryonic ovaries, as well as primordial germ cell-like cells (PGCLCs) induced by pluripotent stem cells (Figure 4) (Hikabe et al., 2016; Morohaku et al., 2016). The culture can be roughly divided into three parts, i.e., *in vitro* differentiation (IVD), *in vitro* growth (IVG), and *in vitro* maturation (IVM) (Hayashi et al., 2017). The first part is hormone-independent, whereas the latter two parts are FSH-dependent processes. IVD is the period from PGCs to secondary follicles, IVG induces antral follicles, and IVM generates MII oocytes. After IVM, MII oocytes can be fertilised by *in vitro* fertilization (IVF) and developed to generate offspring by transplantation into the surrogate mother. Therefore, *in vitro* culture can generate functional oocytes that replicate the entire process of oogenesis in a dish. When PGCLCs are cultured to generate oocytes, somatic cells from the embryonic ovaries are required for maturation. Currently, these somatic cells are also induced from embryonic stem (ES) cells (Figure 4) (Yoshino et al., 2021). Therefore, it has been suggested that oogenesis recaptures only in pluripotent stem cells. Although *in vitro* culture produces mature oocytes, it is not the same as oogenesis *in vivo*. One notable difference is that primordial follicles are not formed *in vitro*, indicating that some environmental factors are not reproduced *in vitro*. In terms of microenvironment, primordial follicles are found in the ovarian cortex, which is an enriched extracellular matrix that maintains the dormant state of primordial follicles (Bochner et al., 2015; Nagamatsu et al., 2019). When the extracellular matrix is digested by enzymes, primordial follicles are activated. However, if digestion of the extracellular matrix is performed under physiological pressure, oocytes remain dormant. This indicates that primordial follicles are under pressure from the extracellular matrix in the ovarian cortex (Nagamatsu et al., 2019). This finding was applied to *in vitro* culture by pressurization, and primordial follicles were successfully induced *in vitro* (Nagamatsu et al., 2019). Furthermore, the ovarian cortex is distant from the blood vessels, indicating that primordial follicles experience hypoxic conditions (Feng et al., 2017); interestingly, hypoxic cultures can also induce primordial follicles *in vitro* (Shimamoto et al., 2019). These findings highlight the importance of understanding the microenvironment and replicating it *in vitro*. Since primordial follicles can be induced *in vitro*, it may be possible to overcome the difficulty of oocyte number decrease with age. Therefore, it is of interest to analyse the changes in vitro-induced primordial follicles due to long-term culture.

## 6 Discussion

Oocytes in primordial follicles are directly related to the preservation of function because they must retain genome stability and developmental potential as reproductive cells, while being maintained in a dormant state for a long time. Age-related changes in oocytes are twofold, qualitative and quantitative functional deterioration. Primordial follicles are involved in both aspects and elucidating their characteristics would lead to a fundamental understanding of age-related changes in oocytes.

### 6.1 Metabolic changes in oocyte aging

FOXO3 plays an essential role in maintaining the quiescent state of oocytes in primordial follicles (Castrillon et al., 2003), but the downstream factors and other gene regulatory networks involved remain unknown. In addition, hypoxic culture revealed that oxidative phosphorylation was suppressed in dormant oocytes in the primordial follicles (Shimamoto et al., 2019). FOXO3 has been reported to regulate energy metabolism in response to stress signals in haematopoietic and tumour cells (Miyamoto et al., 2007; Tothova et al., 2007). The inhibition of oxidative phosphorylation is directly involved in energy metabolism. Specific energy metabolism is necessary for oocytes in primordial follicles to maintain their functions in the dormant state for a long period of time. Suppression of oxidative phosphorylation in primordial follicles suggests a characteristic metabolic pathway for pyruvate; the two pyruvate metabolic pathways are well known: one involves its conversion to acetyl-CoA by pyruvate dehydrogenase and activates the tricarboxylic acid (TCA) cycle, while the other involves conversion to lactate by lactate dehydrogenase (LDH). In the LDH pathway, pyruvate is a possible source of NAD when producing ATP during glycolysis. Research shows that NADH is accumulated in oocytes in primordial follicles (Cinco et al., 2016). Meanwhile, pyruvate also has a metabolic pathway that reacts with glutamate and converts it to α-ketoglutarate (αKG) and alanine. αKG is an intermediate product of the TCA cycle and a coenzyme for many demethylation-related enzymes. It has been reported that a DNA hydroxymethylase ten-eleven translocation, which requires αKG as a coenzyme, works in maintenance of oocytes in primordial follicles (Yu et al., 2013). Therefore, it is important to clarify gene networks and metabolic regulation involved. Based on these findings, it is important to analyse changes of these steady state of oocyte in primordial follicle with aging.

### 6.2 Quality control of oocyte in primordial follicle

As primordial follicles are stored for a long period of time, it is necessary to ensure embryogenesis and genome stability. In addition, it is hypothesized that quality control may occur in primordial follicles. One study reported that mice contained largely deleted mitochondrial DNA (ΔMt-DNA) with intact mitochondrial DNA (Mt-DNA) (Sato et al., 2007). As ΔMt-DNA has a shorter genome, it is replicated more quickly than normal Mt-DNA. Therefore, the rate of ΔMt-DNA increases with age. However, oocytes showed a decrease in the amount of ΔMt-DNA, and offspring contained only wild-type Mt-DNA (Sato et al., 2007). This has been implicated in quality control mechanisms during oocyte maturation. Furthermore, it has recently been reported that FOA, which involves a massive loss of oogonia in the foetal ovary, escapes through checkpoint kinase (CHK) 2 mutation and retrotransposon inactivation (Tharp et al., 2020). Although it has been predicted that FOA guides quality control of oocytes and/or nursing to concentrate organelles, such as mitochondria, the escape from FOA has no effect on developmental potential (Tharp et al., 2020). Although further study is required, it is possible that there is a quality control machinery in oocytes after primordial follicle formation. Indeed, it has reported that p63 acts as a quality control for DNA damage in oocytes in primordial follicles. Whereas p63 remains inactive in oocyte under normal physiological conditions, in the event of p63 activation, pro-apoptotic genes such as Puma and Noxa are induced, resulting in cell death. Consequently, abnormal activation of p63 has been implicated in the onset of POI (Lena et al., 2021).

### 6.3 New perspective to approach oocyte aging

Since the number of primordial follicles decreases with aging, it is difficult to analyse them quantitatively. However, recent developments *in vitro* culture systems have made it possible to induce primordial follicles *in vitro* and to analyse the characteristics and mechanisms of oocyte preservation over a long period of time. For example, using an *in vitro* culture system, it is anticipated that the search for drugs that control the activation of primordial follicles as well as the elucidation of molecular mechanisms through functional gene screening using CRISPR/Cas9. In particular, functional genes also be expected to be used as markers for dormant state of primordial follicles. One of the reasons why it is difficult to analyse the aging changes of primordial follicles *in vivo* is the problem of the lack of marker genes. The dormant state of primordial follicles had been defined by the nuclear localization of FOXO3 in oocytes and morphological changes in granulosa cells. However, with this method, it is necessary to confirm by immunostaining after fixation, and it is difficult to analyse the metabolic state and the comprehensive gene expression. Furthermore, when the primordial follicle numbers decrease with age, detection itself becomes difficult. To solve this situation, it is necessary to identify marker genes that allow detection of dormant state of primordial follicles. Once marker genes are identified, creating a mouse that expresses fluorescent proteins under the control of these genes would make it possible to detect dormant state of primordial follicles in live cells, and it would also be possible to isolate them using flow cytometer. Furthermore, in addition to genes, the identification of important factors such as lipids can be expected. Whereas oocytes have a larger volume and store more substances than somatic cells, it is not well understood which nucleic lipids are involved in the aging, especially of primordial follicles (Table 1). In this respect, further development is expected in the future Komatsu et al. (2006); Lapa et al. (2011); Kujjo and Perez (2012); Mok et al. (2016); Li et al. (2019).

**TABLE 1:** Lipids involved in oocyte aging.

Lipid	Characteristic	References
Lysophosphatidic acid	Promote meiotic maturation	Komatsu et al. (2006)
Polyunsaturated fatty acid	Related to oocyte quality	Lapa et al. (2011)
Ceramid	Decrease with age	Kujjo and Perez (2012)
Phosphatidic acid	Decrease with age	Mok et al. (2016)
Phosphatidylinositol	Decrease with age	Mok et al. (2016)
Phosphatidylserine	Decrease with age	Mok et al. (2016)
Lysophoshatidylserine	Decrease with age	Mok et al. (2016)
Sphingolipid	Biomarkers for PCOS	Li et al. (2019)

Efficient use of *in vitro* culture systems is expected to dramatically advance the understanding of the aging of primordial follicles and oocyte growth. It is willing to develop the techniques for controlling the functional decline caused by oocyte aging in the future.
